# Revascularization and angiogenesis for bone bioengineering in the craniofacial region: a review

**DOI:** 10.1007/s10856-023-06730-6

**Published:** 2023-05-30

**Authors:** Randa AL-Fotawi, Waleed Fallatah

**Affiliations:** 1 Oral and Maxillofacial Dept. Dental Faculty, King Saud University, Riyadh, 11451 Saudi Arabia; 2 Medical collage, Alfaisal University, Riyadh, Saudi Arabia

**Keywords:** Revascularization, Reconstructive surgery, Bioengineering, Ischemia–reperfusion injuries, Neovascularization, Mesenchymal stromal cells

## Abstract

The revascularization of grafted tissues is a complicated and non-straightforward process, which makes it challenging to perform reconstructive surgery for critical-sized bone defects. This challenge is combined with the low vascularity that results from radiotherapy. This low vascularity could result from ischemia–reperfusion injuries, also known as ischemia which may happen upon grafting. Ischemia may affect the hard tissue during reconstruction, and this can often cause resorption, infections, disfigurement, and malunion. This paper therefore reviews the clinical and experimental application of procedures that were employed to improve the reconstructive surgery process, which would ensure that the vascularity of the tissue is maintained or enhanced. It also presents the key strategies that are implemented to perform tissue engineering within the grafted sites aiming to optimize the microenvironment and to enhance the overall process of neovascularization and angiogenesis. This review reveals that the current strategies, according to the literature, are the seeding of the mature and progenitor cells, use of extracellular matrix (ECM), co-culturing of osteoblasts with the ECM, growth factors and the use of microcapillaries incorporated into the scaffold design. However, due to the unstable and regression-prone capillary structures in bone constructs, further research focusing on creating long-lasting and stable blood vessels is required.

**Graphical Abstract:**

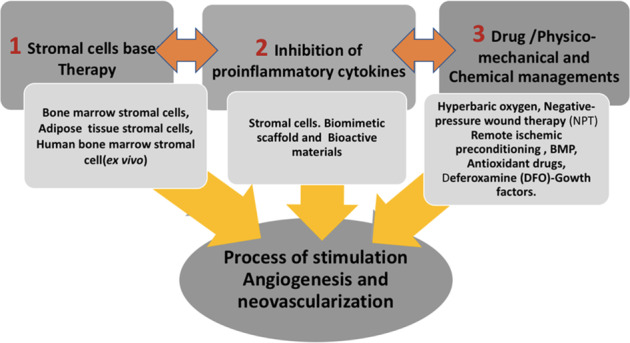

## Introduction

Reconstructive surgery for critical-sized defects is still considered a challenge due to poor tissue perfusion, called ischemic–reperfusion injury or ischemia [1]. Bone healing can still occur with the initial transient decrease in blood flow, and angiogenesis and revascularization are necessary for normal bone healing [2]. Tissue necrosis of the flap can affect hard tissue-like bone during reconstruction, resulting in infection, resorption, malunion, and disfigurement.

Angiogenesis has been defined as the formation of new vessels from pre-existing vascular networks through the proliferation and migration of differentiated endothelial cells [3]. Whereas neovascularization occurs in the case of inflammation, malignancy, and ischemia [4]. Neovascularization was defined in more detail by Velazque, who stated that local factors at the site of inflammation or tissue injuries stimulate angiogenesis from the existing vasculature and promote the recruitment of circulating bone marrow-derived endothelial precursor cells (EPCs) that contribute to vasculogenesis [5].

Physiologic angiogenesis passes through three stages: stimulus, initiation, and resolution. The stimulus can trigger an angiogenesis cascade. In the initiation phase, an increase in endothelial cell-derived growth factors (GFs), such as vascular endothelial growth factor A (VEGFA) and fibroblast growth factor (FGF), cause vessel destabilization and initiate vessel sprouting and endothelial cell proliferation. Matrix metalloproteinases (MMPs) facilitate the remodeling of the extracellular matrix (ECM) and increase the biological availability of ECM-sequestered growth factors. Finally, the resolution phase starts once the new vessels are formed and perfusion is established in which VEGF levels decline with a rise in platelet-derived growth factor (PDGF), angiopoietins (Ang), and transforming growth factor-β1 (TGFβ1) (Chung et al. 2010) [6]

The process of neovascularization in adult involve manly angiogenesis and percentage of vasculogenesis (3.5% and 25%) in a single microenvironment [7, 8]. The process of neovascularization starts under the influence of number of factors including hypoxia, hypoglycemia, mechanical stresses, inflammation and genetic mutations related to cancer or disease.

In General, it stimulates migration of Vascular Endothelial cells (VEC) into extracellular matrix, then assembling network structures. The role of other supporting cells like pericytes, vascular mural cells are also important in keeping vessel stability, mechanical function and response to physiological changes.

In bone regeneration two biological mechanisms were proposed in literature, for direct bone healing, VEC and perivascular mesenchymal cells provide the osteoprogenitor cells that differentiate into osteoblasts and create lamellar bone to achieve complete union during direct fracture healing. On other hand, vascular endothelial growth factor (VEGF) is considered a key regulator of vascular regeneration in endochondral fracture healing. More vascularization and blood perfusion related to the greater inflammatory response and higher angiogenic cytokine secretion in case of indirect fractur healing may occur [9].

Neovascularization, and osteogenesis are integral to acute fracture healing and have been an important component of studies in bone tissue engineering [10], which has been defined as “The application of the principle and methods of engineering and the life sciences toward the development of biological substitution to restore, maintain or improve functions” [11]. Cultivating vascularity and inducing robust neovascularization is the key of success in reconstructive surgery. Cell-based therapy using biomimetic scaffolds that provide microenvironmental and inductive extra-cellular cues and that can preserve host cell properties when implanted in vivo are necessary for optimum tissue engineering.

**Fig. 1:**
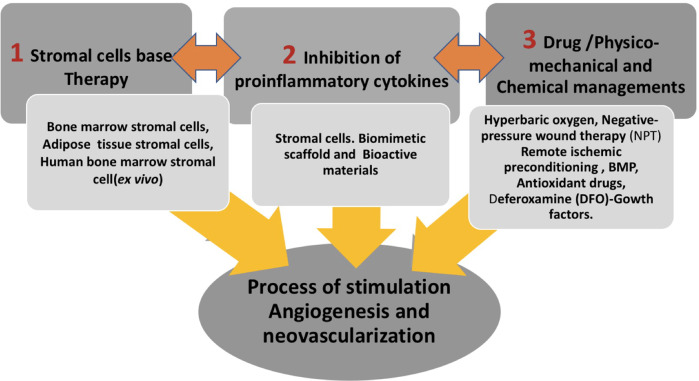
Schematic illustration summarizing the three main strategies to stimulate neovascularization and angiogenesis to overcome the problem of ischemia while grafting. 1. Using cell-based therapy to sustain the angiogenic process through the secretion of vascular growth and inhibition of proinflammatory mediators. 2. Inhibition of proinflammatory mediators can occur through the use of different bioactive scaffolds and the use of biomimetic scaffolds. 3. The use of medications and mechanical and physico-chemical stimuli were proven to stimulate and sustain the process of neovascularization or to prevent ischemic perfusion injury

Different methods have been proposed in the literature to overcome the problem of ischemic reperfusion injuries at the clinical and pre-clinical levels and were included in this review (see Fig. 1). This review discusses previous and current trends regarding the induction of angiogenesis and neovascularization using tissue engineering at the clinical and ex vivo levels.

## Methodology

Search methodology: This research was based on the Population Intervention Comparison Outcome Study design (PICOS) format. The PubMed, Web of Science, Cochrane, and Ovid databases were used for the literature search. The inclusion and exclusion criteria are listed in Table 1. Clinical human trials, case series, and case reports that investigated the role of tissue engineering to overcome the problem of vascularity in repairing critical-sized bone defects using cell-based therapy, bone morphogenic proteins, or physiochemical stimulation. Summary for the selected papers based on the inclusion criteria and the strategy used to induce neo-vascularization are highlighted (Table 2).

**Table 1:** The inclusion and exclusion criteria for the selected paper in the review

Inclusion Criteria	Exclusion Criteria
Clinical controlled trials, case series, case reports for flap design or in vivo experiments showing techniques using tissue engineering to overcome the problem of ischemic reperfusion injuries or to enhance revascularization.	Exclude conventional strategy of bone regeneration small bone defect or 3 wall defect.
Reports that used either cell therapy, growth factor BMP, or physiochemical tissue stimulation aiming to stimulate osteogenesis and angiogenesis.	Papers limited to reports using scaffolds only with no clear study objective to stimulate osteogenesis and angiogenesis.
Up to date review articles or experiments explain in depth the biology and mechanism of neovascularization or/and new approaches of tissue engineering focused primarily on bone regeneration	Up to date review articles or experiments explain in depth the biology and mechanism of neovascularization or/and new approaches of tissue engineering other than bone regeneration
Long follow up(more than 12 month) including data presented or/and reported surgical re-entry for implant placement dental rehabilitation(even less than 12 months).	Short follow up(less than 12 month) with no surgical re -entry is presented for dental rehabilitation
Pervious works were duly acknowledged.	Previous works not acknowledged.
Study conclusions justify the authors’ objectives.	Study conclusion did not justify the authors’ objectives.

**Table 2:** Summary of the selected research papers and the strategy used for neo-vascularization for bone regeneration on critical-sized defect

Neovascularization Strategy	Authors	Constructs Design	The Study Type	The Study Subject
Skeletal Muscle	Warnke et al. [15]	Use Skeletal Muscle As Bioreactor/MSCs or BMP	Case report	Human mandible
	Heliotis et al. [16]		Case report	Human mandible
	Alfotawi et al. [20]	Use skeletal muscle(flap,or graft as a Biomimetic scaffold/MSC and BMP	Single cohort	Mandible Rabbits
	Alfotawi et al. [19]		Controlled experiment	Cranial Rat
	Porzionato et al. [24]	Use Decellurized skeletal muscle(graft) as a Biomimetic scaffold/SCPC and MSCS		
	Alfotawi et al. [25]		Controlled experiment	Cranial mice
Scaffold designs and fabrication	Zhang et al. [36] Makhoul [35]	fabricated vasculature inside the 3D scaffolds USING silicate bioceramic OR polymer nanofibers	Controlled experiment	the rabbit radius by, critical size defects
	Liu et al. [30] Duan et al. [31]	3D-printed scaffold PCL/HA *with interconnected hollow-pipe structures*	Controlled experiment	rat cranial defect femoral epicondyle
	Yan et al. [32]	deferoxamine (DFO)-loaded PCL scaffolds	Controlled experiment	Rat large bone defect
	Wang et al. [33]	angiogenic peptide and osteogenic peptide were-loaded (TCP/PLGA)	Controlled experiment	Rat cranial defect
	Lee et al. [34]	polycaprolactone (L-lactide-co-ε-caprolactone) (PLCL), and a hydrogel made of decellularized adipose tissue extracellular matrix (ECM) and collagen	Controlled experiment	Rat sub cutaneous tissue
Bone Morphogenic Proteins BMP	Moghadam et al. [37]	BMP	Case report	Human mandible
	Ferreti and Ripamotnit [38]	BMP2	case series study	Human mandible
	Marx et al. [38]	BMP2/PRP	Control Trial	Human mandible
	Chao et al. [40]	BMP2/Stromal cells	Case report	Human mandible
	Herford et al. [41]	BMP2	case series study	Human mandible
	Cicciu et al [42]	BMP2	Case report	Human mandible
	Ayoub et al. [43]	BMP-7	case series study	Human Maxilla
Cells Based Therapy	Kioshita et al. [49].	Bone marrow stromal cells(	Experiment/ 2Case report	Human Mandible
	Lee et al. [50]	Differentiated MSC Mesenchymal Stromal Cells	Case report	Human Mandible
	Hernadnems-Afaro et al. [51]	Bone Marrow concentrate/BMP7	Case Report	Human mandible
	Zamiri et al. [52]	MSCs	case series study	Human mandible
	Kim et al. [53]	MSCS	Case report	Human mandible
	Wolff et al. [54]	Adipose Stromal Cells	Case report	Human mandible
	Sandor et al. [55]	ASCs/BMP-2	Case report	Human mandible

## Which patients require bone/tissue engineering?

There are particular patient categories that require tissue and bone engineering because of the nature of their medical and health conditions. The first category of patients among these are patients who experience chronically upregulated immune systems. These conditions are experienced in patients such as those with rheumatoid arthritis, systemic lupus erythematosus, or diabetes. Another group is patients who have experienced acute dysregulation of their immune systems. One example of this can be seen in polytrauma and sepsis patients. Some of the risk factors for nonunion include smoking, patients with old age, diabetic patients, and patients using non-steroidal anti-inflammatory medications [12, 13].

## Literature-reported methods of avoiding ischemia–reperfusion injuries

### Outline of the clinical and experimental works for the induction of angiogenesis and revascularization by utilizing skeletal muscles/active biomaterial

Evidence in the literature shows that when muscles are exposed to osteogenic stimuli, such as bone matrix substitutes, or to Bone morphogenic proteins, they develop some intrinsic osteogenic properties that are crucial in developing and facilitate the process of bone formation [14–18]. Previous studies have demonstrated the colonization of the endothelial cells into muscle tissues when used as scaffolds make it possible to reestablished a three-dimensional vascular network after the implantation process [19]. The aim of the clinical case reports and ex vivo experiments was to target the use of the skeletal muscles as a bioreactor. These case studies have been discussed [15, 16, 18–20] in vivo experiments. For these reasons, most of the case studies have suggested the use of the skeletal muscles as a biomimetic supporting scaffold in order to facilitate the bone formation process, which also provides the much-needed vascularity. Summary for the role of biomimetic scaffolds is critical in the maintenance of stem cell properties and the possibility of reprogramming their commitment. They support and maintain either a differentiated or an undifferentiated status of the mesenchymal stromal cells (MSCs). There are different elements that could affect the cells; these are oxygen tension, glucose concentration, growth factors, and the physiochemical nature of the environment including the pH and ionic strength (e.g., Ca^2+^ concentration) [21]. In addition, the use of biomimetic scaffolds was proven to allow the modulation of the immune environment in favor of regenerative processes and suppression of tissue degeneration [22].

The clinical case reported by Heilotis et al. included a case of a 60-year-old patient who was awaiting hemimandibulectomy following radiotherapy. The procedure involved the adjustment of the three blocks of hydroxyapatite HA to resemble the shape of the ramus and mandibular body onto Pectoralis muscle for a 15-weeks period. These costructs were infused with BMP-7, then implemented within the mandibular defect. The patient later developed the MRASA [16].

The study by Warnke et al. provided the case of a clinical case study in which rhBMP-7 was seeded with mesenchymal stromal cells (BMSCs). These were then used together with a titanium mesh tray to establish an external scaffold and were added to a degradable polylactide scaffold. In this report, the prepared construct was implanted into the latissimus dorsi muscle of the patient over a period of 7 weeks. However, in this case, the patient died from cardiac arrest within 15 months of the implantation [15].

**Fig. 2:**
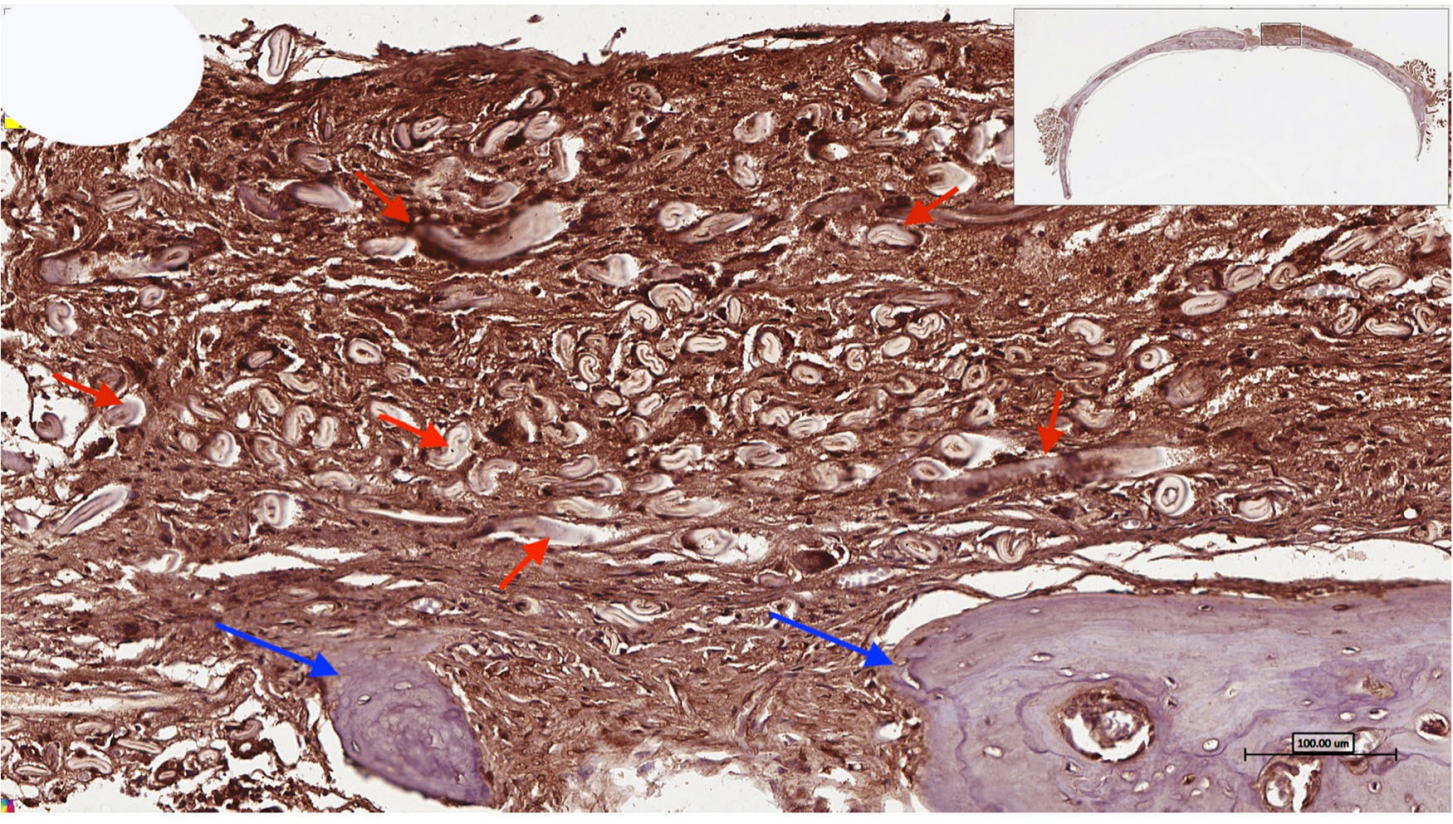
Higher-magnification image of the center of the bony defect showing the use of treated, decellularized muscle for the reconstruction of numerous bone spicules (blue arrows) surrounded by connective tissue that shows the positive intake of osteopontin (OPN) immunostaining

Further clinical experimentations suggest the use of In vivo osteogenic properties for the grafting of skeletal muscles. Such experimentation shows that novel strategies can be implemented to facilitate effective bone regeneration [19, 20, 23]. For example, more recently, it has been established that decellularized skeletal muscles (Fig. 2) can be utilized for bone augmentation to facilitate regeneration and to restore the contour and volume of the lost bone. The experiment also included histological assessment that showed the formation of neovascularization which showed a potential graft strategy for patient presented with massive bone resorption or critical-sized defects [24, 25].

### Jaw reconstruction using bone morphogenic proteins (BMPs), growth factors, and scaffolds

BMPs are one of the vital elements of the vascular system. This vitality was previously established in experimental studies that involved both rat models and human vascular disorders, as shown by ref. [26]. BMPs play crucial roles in the process of endothelial cell differentiation, as well as during the process of venous/arterial and lymphatic specification. Goumans et al., in their experiment, demonstrated clearly that the signaling process using bone morphogenetic proteins plays a critical role in regulating vascular functioning [26].

Additionally, the role of bioactive third-generation scaffolds was also highlighted in the literature. Zhai et al. reported that silica (Si) ions that are present in active bioceramics, such as silica calcium phosphate cement (SCPC), stimulated the human umbilical vein endothelial cells to highly express vascular endothelial growth factors (VEGFs) and subsequently activated kinase domain insert receptors by themselves to initiate the angiogenesis pathway [27]. Similarly, Deng et al. reported the addition of interferon-gamma I to calcium silica oxide (CaSiO_3_) added to beta tricalcium phosphate (β-TCP) 3D-printed scaffolds, indicating the effectiveness of the dual release of IFN-γ and Si from scaffolds in modulating macrophage polarization and promoting vascular ingrowth [28].

Various emerging strategies by which to enhance vascularization in bone grafts have been proposed in the literature using three-dimensional (3D) printed scaffolds. Some used a simple implantation of growth factors, which is known to enhance vascularization, while others were more sophisticated, using fabricated vasculature inside the 3D scaffolds in vitro, which were then directly anastomosed or micro-surgically connected once implanted in vivo [29]. The following are examples of 3D-treated scaffolds for bone regeneration: VEGF was conjugated into 3D-fabricated polycaprolactone (PCL) and hydroxyapatite (HA), resulting in a 3D-printed scaffold PCL/HA with interconnected hollow-pipe structures [30, 31]. In 2019, Yan reported the use of deferoxamine (DFO)-loaded PCL scaffolds. Vascularized 3D-printed scaffolds have been used for the promotion of bone regeneration [32]. Additionally, angiogenic peptide and osteogenic peptide were loaded into tricalcium phosphate/polylactic glycolic acid (TCP/PLGA) composite scaffolds were constructed via cryogenic 3D printing and subsequent hydrogel coating [33]. Moreover, a 3D-printed scaffold was made of a highly elastic polycaprolactone (L-lactide-co-ε-caprolactone) (PLCL), and a hydrogel was made with decellularized adipose tissue extracellular matrix (ECM) and collagen. The 3D-printed scaffolds were subcutaneously implanted in rats for in vivo experiments [34]. The latter reported a higher vascularization rate when compared with the use of PLCL for bone defects in an animal model.

Finally, artificial microvessels were implemented inside a 3D-printed scaffold, which was implanted in vivo [35, 36]. Another example is the model constructed by Zhang et al., which was made of silicate bioceramic scaffolds with hollow struts, and a successful result was reported [36].

Clinically, Moghadam et al. studied the use of allogeneic bone, which was infused with BMP together with their reconstruction plate. This was implemented in one patient in whom the ameloblastoma of the mandible was also removed [37]. In the study by Ferreti and Ripamotnit, 13 patients were involved in an experiment in which collagen was used as carrier for BMP2, aiming at solving the challenge of mandibular continuity defect reconstruction [38]. In the clinical by Marx et al. 40 patients were involved who require mandibular reconstruction. A composite grafts was made using rhBMP-2 added to crushed cancellous freeze-dried allogeneic bone (CCFDAB), and platelet-rich plasma (PRP) was used for experimental group, whereas the control group was grafted using autogenous graft. All the successful autogenous grafts regenerated sufficient bone for implant restoration compared to 97.4% of the composite grafts [39].

The study by Chao et al. also reported a case of a 12 cm ossifying fibroma reported in a 9-year-old male patient. These cases were handled using 36-weeks intervention plan that involved the use of a reconstruction plate, a collagen carrier for rhBMP-2 and stem cell concentration, which were all included in the Tricalcium phosphate/hydroxyapatitte (TCP + HA) [40]. A further study by Herford et al. later reported an experiment that showed successful reconstruction. This process was successfully undertaken among 12 patients using type I collagen and the BMP-2 [41].

Cicciu et al. also studied the case of reconstruction within a patient who experienced an en-bloc resection in the dentinogenic ghost cell. In this process, the medical practitioners used an inferior titanium plate, and then added rhBMP-2 to the Collagen Sponge with the help of an allograft. After a period of 9 months, they then removed the titanium mesh, and they were able to insert dental implants in the patient [42]. An additional study conducted by Ayoub et al. also reported the reconstruction of an alveolar cleft using 3.5 mg of the recombinant human bone morphogenic protein-7 (rhBMP-7). This study, which involved at least 11 patients, utilized the type I collagen carrier and similarly presented promising results regarding the use of this reconstruction method [43].

Despite the existence of the many success stories that have led to significant milestones in reconstruction, it is important to note that, in the cases where only BMP was used, the studies reported a 13.9% rate of failure. This is especially significant when reconstruction is carried out involving large bone defects and defects located mainly in the maxillofacial region of the human body [44].

### Mesenchymal stromal cell (MSC)-based therapy

The rationale for using cell-based therapy is based on two main factors: in addition to providing a multipotency of the cell to transform into a different cell language, cell therapy also presents the opportunity for cells to stimulate or suppress the immune system. The suppression of the enhancement of the immune system follows the varied intervention of macrophages, T and B cells, dendritic cells, and natural killer cells. Additionally, MSCs may be considered therapeutic agents in inflammation treatment (Jin 2022) [45]. On other hand, stromal cells are capable of sustaining the angiogenic process through the secretion of vascular growth- and inflammation-promoting factors, granulocyte colony-stimulating factors (GCSF), and interleukin1β (IL-1β,) resulting in a failure to resolve the angiogenesis cascade [6].

Understanding the impacts of cell theory is vital for the treatment of certain conditions, such as patients who have experienced acute dysregulation of their immune systems. Such dysregulation is normally seen in sepsis or polytrauma cases[46]. Other cases are also experienced by patients with systemic lupus erythematosus, rheumatoid arthritis, or diabetes [46]. Several researchers have also reported the significance of employing cell therapy in such processes, especially for neovascularization and angiogenesis. This takes place after modifying, pre-conditioning, and even genetically inducing the seeded cell [47, 48].

Several clinical report cases have also been conducted utilizing cell-based therapy. The first of these was the report by Kioshita et al. This study used A-poly L-lactic acide PLLA mesh, which was designed in the shape of an alveolar bridge. In this case, the mesh was used to reconstruct the mandible areas with the help of the autogenous particulate cancellous bone together with the whole bone marrow aspirate. The cells were left intact without separation. In two of the clinical cases, the augmentation was carried out through the use of hydroxyapatite. Overall, the clinical report showed significantly positive outcomes for reconstruction defects in the mandibles [49].

The second case report in this category was conducted by Lee et al., and this report involved the case of a single patient in whom the resected jaw was frozen then the reconstruction was made using differentiated MSCS and fibrin glue. Guided bone regeneration was used to guide the overall process of the bone generation techniques. Subsequently, the report was concluded with the use of a dental implant to replace the dental structure [50]. A similar technique was also studied in each clinical case report by Hernadnems-Afaro et al. In this case, the report involved a 33-year-old patient who presented with a complication of recurrent ameloblastoma, and with a titanium mesh. In this case, the patient was infused with 2 g of rBMP-7 and 5 mL of bone marrow concentrate. After nine months, a surgical re-entry was performed for an implant placement [51].

In the study by Zamiri et al., three serious cases were reported in which the patients all had mandibular defects of a critical size. In these cases, autogenous MSCs and mandibular bone were implemented to perform the reconstruction. Among these cases, one reported failure, while the others reported success [52]. In a similar study, the case discussed by Kim et al. involved mandibular reconstruction, which was conducted using autologous human bone marrow stem cells (BMSCs). This process was combined with autogenous bone grafting to restore mandibular defect in patient with plexiform ameloblastoma. The grafting was performed for this patient 6 months after the initial operations, and this also allowed planning for the implant. The report, therefore, stated that the use of BMSCs is a viable alternative to the previous method of autologous bone grafting [53].

Another case study was also presented, in which three patients were grafted using adipose stem cells (ASCs) seeded into a β-TCP scaffold. As reported by Wolff et al., these stem cells were then infused with the BMP-2 [54]. During the same period, a study by Sandor et al. also reported a case of successful reconstruction that was conducted using a reconstruction plate with ASCs/β-TCP and BMP-2. Ten months after the reconstruction surgery, six endosseous dental implants were inserted in the regenerated bone for dental rehabilitation [55].

## Conclusions

This review illustrates different strategies by which to overcome the problem of ischemic–reperfusion injury upon grafting critical-sized bone defects. The current trends described in the literature are the seeding of the mature and progenitor cells into extracellular matrix (ECM), co-culturing of osteoblasts with the ECM, growth factors, and the use of microcapillaries into scaffold design. However, due to the unstable and regression-prone capillary structures in bone constructs, further research focusing on creating long-lasting and stable blood vessels and the incorporation of vasculature into individual compartments is required.

### Data sharing

Not applicable to this article as no datasets were generated or analyzed during the current study

## Abbreviations

EC: extracellular
ECM: extra cellular matrix
GF: growth factors
VEGFA: vascular endothelial growth factors
FGF: fibroblast growth factor
MMP: Matrix metalloproteinases
PDGF: platelet-derived growth factor
Ang: angiopoietins
FGEß1: transforming growth factor beta-1
MSCs: mesenchymal stromal cells
BMP: bone morphogenic protein
SCPC: silica calcium phosphate cement
ß-TCP: beta tricalcium phosphate
CaSiO: calcium silica oxide
3D: three-dimensional
PCL: poly caprolactone
DFO: deferoxamine
HA: hydroxyapatite
PLCL: L-LACTIDE-CO-Œ-Ca prolactone
rhBMP-7: recombinant human bone morphogenic protein
GCSF: granulocyte colony-stimulating factor
IL-1ß: interleukin 1 beta
VEC: vascular endothelial cells
